# Effects of irrigation and nitrogen fertilization on mitigating salt-induced Na^+^ toxicity and sustaining sea rice growth

**DOI:** 10.1515/biol-2022-0492

**Published:** 2022-09-14

**Authors:** Li Jin, Fan Xiao-lin, Zhu Yin-ling, Rao Gang-shun, Chen Ri-sheng, Duan Ting-ting

**Affiliations:** Guangdong Ocean University, Zhanjiang, Guangdong 524088, China; South China Agricultural University/Environment Friendly Fertilizer Engineering Technology Research Center, Guangzhou, Guangdong 510642, China

**Keywords:** sea rice, irrigation, nitrogen fertilization, saline-sodic soil, sodium ions, yield

## Abstract

This study investigated the effects of irrigation and nitrogen (N) fertilization on mitigating salt-induced Na^+^ toxicity and sustaining sea rice growth for perfecting irrigation and fertilization of sea rice. Three irrigation methods (submerged irrigation, intermittent irrigation, and controlled irrigation), three kinds of N fertilizers (urea, controlled release urea, and mixed N fertilizer), and control treatment without NaCl were set up in a pot experiment of sea rice with NaCl stress. The electrical conductivity in root layer soil of treatment with mixed N fertilizer and intermittent irrigation decreased slowly with the growth of rice and was significantly smaller than that of other treatments with NaCl. The Na^+^ content in sea rice of intermittent irrigation was the least, and that of submerged irrigation was significantly smaller than that of controlled irrigation, but the K^+^ and Ca^2+^ contents of three irrigation treatments were opposite to the Na^+^ content. The Na^+^ content of treatment with mixed N fertilizer and intermittent irrigation was the lowest, while the K^+^, Ca^2+^, and Mg^2+^ contents of mixed N fertilizer and intermittent irrigation were the highest in treatments with NaCl. The cell membrane permeability and malondialdehyde contents of rice leaves of mixed N fertilizer and intermittent irrigation were significantly smaller than those of other treatments with NaCl. The rice yield of mixed N fertilizer was significantly greater than that of urea and controlled release urea, and that of mixed N fertilizer and intermittent irrigation was increased by 104, 108, 277, 300, and 334% compared with mixed N fertilizer and submerged irrigation, urea and intermittent irrigation, urea and submerged irrigation, controlled release urea and intermittent irrigation, and controlled release urea and submerged irrigation, respectively. Therefore, the treatment of mixed N fertilizer and intermittent irrigation is worth recommending for being used for planting sea rice on coastal saline-sodic soil.

## 1 Introduction

Presently, soil salinity is an enormous problem for world agriculture, and about 20% of the world’s arable land is affected by salinity [1]. It has been estimated that more than 50% of the arable land would be salinized by the year 2050 [2]. There are 100 million hectare saline land [3] and 2.34 million hectare beaches [4] in China. Saline land is a rare land reserve resource with great potential for comprehensive utilization, but salt stress is one of the main adverse factors to crop yield [5]. In recent years, the use of salt-tolerant rice (sea rice) as a pioneer crop in salty environments has become an effective measure for the restoration and use of beaches and saline lands and provide a new way to solve the problem of food security [3].

Soil salinization is usually caused by the accumulation of sodium chloride (NaCl) [6]. The growth and development of plants are slow under NaCl stress, and its metabolic ability is inhibited, which may cause wilting and death of plant [7]. The irrational fertilization and irrigation will lead to the accumulation of salt in root zoon soil [8], and a large amount of salt ions such as sodium (Na^+^) will be accumulated in rice, breaking the ion balance in plant, resulting in its physiological metabolism disorder and water imbalance. This is averse to the growth and development of rice and reducing the yield of rice [9]. Therefore, the reasonable irrigation and fertilization are important measures to reduce salt content in saline soil and increase crop yield [8]. Rice is one of the crops that demand for most nitrogen fertilizer, and the application of nitrogen fertilizer plays a decisive role in the yield of rice [10]. The use of controlled-release nitrogen fertilizer can mitigate salt accumulation in soil due to the slow release of nutrient from fertilizer, which is beneficial for crops to absorb nitrogen, and the nitrogen is conducive to maintaining normal physiological and metabolic functions of rice [11]. Na^+^ entry to rice is inhibited and Na^+^ efflux from rice is promoted by rice selectively absorbing potassium (K^+^), calcium (Ca^2+^), and magnesium (Mg^2+^) ions, and high K^+^/Na^+^, Ca^2+^/Na^+^, and Mg^2+^/Na^+^ are maintained, thereby mitigating, or avoiding salt-induced Na^+^ toxicity for rice [12].

Many studies have shown that the reasonable irrigation and fertilization can control soil salinity and increase rice yield in saline-alkali soil [8,13]. However, the research about sea rice in coastal saline areas is mostly focused on the breeding of salt-tolerant rice varieties, and the lack of irrigation and N fertilization technology of sea rice. Therefore, the main objective of this study is to determine and analyze the effects of different N fertilizers (urea and controlled release urea, etc.) and irrigation methods [submerged irrigation, intermittent irrigation, and controlled irrigation] on the nitrogen uptake, ions (Na^+^, K^+^, Ca^2+^, and Mg^2+^) contents, cell membrane permeability (CMP) and malondialdehyde (MDA) contents, growth, and yield of sea rice under high salt stress (1% NaCl). The effects of irrigation and N fertilization on mitigating salt-induced Na^+^ toxicity and sustaining sea rice growth were studied to optimize irrigation and fertilization of sea rice and provide scientific basis for the correlation study in future. Moreover, we tried to reveal the mechanism of irrigation and N fertilization to mitigate Na^+^ toxicity of sea rice in this study and provide theoretical basis for planting sea rice in coastal saline land.

## 2 Materials and methods

### 2.1 Experimental materials

Test soil: The root layer soil (0–25 cm) was taken from the paddy field of Guangdong Ocean University. The soil pH, bulk density, and organic matter was 6.33, 1.31 g/cm^3^, and 16.08 g/kg, respectively. Available nitrogen, phosphorus and potassium were 63.43, 25.51, and 41.53 mg/kg, respectively. The soil was passed through a sieve (2.00 mm) after air-drying and crushing, and fully mixed with NaCl (10 g NaCl/1 kg soil), and then 32 kg mixed soil was packed into a barrel [inner diameter 28 cm and height 50 cm].

Test crops: The “sea rice 86” (HR86401) provided by Prof. Risheng Chen’s group from Guangdong Ocean University was used as the test rice. It was a salt-tolerant rice that can grow normally on coastal saline soil under 0.6% salinity content, and its root was mainly distributed within 25 cm of soil surface. The rice grains were cultivated in a sterile incubator to three true leaves stage, and seedlings with uniform size were selected and transplanted into the barrel.

Test fertilizer: N fertilizer was provided by Environmentally Friendly Fertilizer Engineering Technology Research Center in Guangdong, including controlled release urea (N ≥ 43.0%, vegetable oil coated urea, 3–4 months validity period), urea (N ≥ 46.0%), mixed N fertilizer made by mixing 30% N urea and 70% N-controlled release urea. Phosphate fertilizer is single superphosphate (P_2_O_5_ ≥ 16%). Potash fertilizer is potassium chloride (K_2_O ≥ 60%).

### 2.2 Study design

The experiment was conducted in the glass greenhouse of Guangdong Ocean University from November 2020 to March 2021. A two-factor split zone design was adopted, the first factor is different irrigation methods, namely, submerged irrigation (S), intermittent irrigation (I), and controlled irrigation (C). The second factor is three kinds of nitrogen fertilizers, which are urea (U), controlled release urea (R), and mixed N fertilizer (M). The nitrogen, phosphorus, and potassium fertilizers of each treatment are mixed with the soil as a base fertilizer and applied at one time. The fertilization rates of treatments were all 0.58 g N, 0.39 g P_2_O_5_, and 0.52 g K_2_O, that is, 1.26 g urea, 1.37 g controlled release urea, 1.32 g mixed N fertilizer, 2.40 g single superphosphate, and 0.86 g potassium chloride per barrel were applied according to the treatment design. In addition, a submerged irrigation and applied urea treatment without adding NaCl was used as a control treatment (CK). A total of ten treatments, each treatment repeated five barrels, and six seedlings were planted in each barrel.

### 2.3 Experiment method

Irrigation method: The S treatment was to keep a 2 cm water layer in the barrel. The I treatment was to stop the irrigation after watering up to the 2 cm water layer, and the water surface was naturally dried before watering, cyclically. The C treatment was continuous precision irrigation and kept 80–100% of soil field capacity by weighing.

#### 2.3.1 Collection of soil and plant samples

The sea rice seedlings were transplanted in November 2020, and the first soil samples were collected at the middle of rice green-returning stage (December 2020). A soil drill was used to collect the root layer soil (0–25 cm) in the barrel and repeat the collection for 5 times (5 barrels/treatment). After removing stones and roots from the soil samples, all soils were passed through a 2 mm sieve and air dried for testing. The second soil and first plant samples were collected at tillering stage of rice (January 2021). Measuring the fresh weight of plant samples was done at first and it was dried at 75°C to a constant weight after curing [105°C, 30 min], then it was weighed again to estimate their dry weight. It was then stored for testing after crushing. The third soil and second plant samples were collected at the middle of the rice booting stage (January 2021). The fourth soil and third plant samples were collected at the mature stage of rice (March 2021) and the yield of rice was measured. The height of sea rice was measured every week.

#### 2.3.2 Determination of plants and soil samples

German STEP soil salinity detector (PNT300 type) was used to regularly check the electrical conductivity (EC) of root layer soil (0–25 cm) in the barrel [14]. After the plant samples were digested with H_2_SO_4_–H_2_O_2_, the nitrogen content was determined with full automatic Azotometer (Shanghai Yihong NKY6120) [14], and atomic absorption spectrophotometer [Japan Shimadzu AA-7000] was used to determine the Na^+^, K^+^, Ca^2+^, and Mg^2+^ contents of single plant [14]. The CMP and MDA contents of rice leaves were measured by the methods of Shunyu Xiang and Kuichao Qu [15,16]. Count the number of effective panicles per pot, the number of grains per panicle, the grain ripening percentage, and the thousand-grain weight of rice to calculate the rice yield per pot.

\text{Yield (g/pot)}=\frac{E\hspace{.25em}\text{(piece/pot)}\times G\hspace{.25em}\text{(piece/pot)}\times P\hspace{.25em}\text{(\%)}\times W\hspace{.25em}\text{(g)}}{\text{1,000}\times \text{100}},]

where *E* stands for effective panicle number (piece/pot), *G* stands for grain number per panicle (piece/pot), *P* stands for grain ripening percentage (%), and *W* stands for thousand-grain weight (g).

### 2.4 Statistical analyses

The mean value, standard deviation, and standard error of date were calculated using Excel 2007 software and analyzed in SPSS 22.0 software for data processing. Analyses of variance (ANOVA) and post-hoc LSD test were used to compare individual mean values. SPSS (version 16) and Sigma Plot (version 11) were used for all statistical analyses and graph preparation, respectively.

## 3 Results

### 3.1 Dynamics of EC in root layer soil under different irrigation and N fertilization

**Figure 1:**
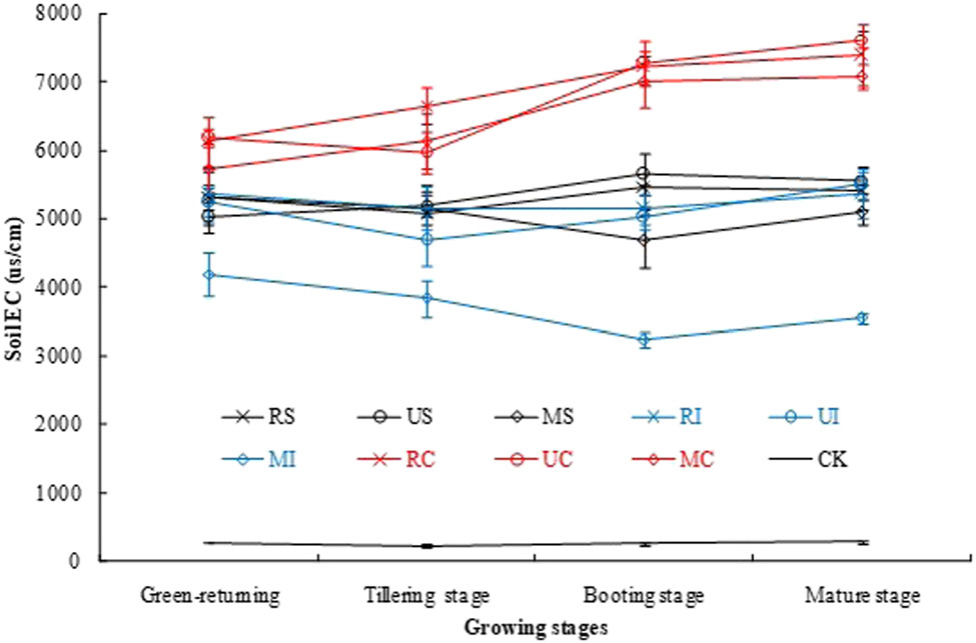
Soil EC under different irrigation and N fertilization treatments.

As shown in Figure 1, the different irrigation and N fertilization treatments have significant effects on root zone soil EC. The EC of C treatments [RC, UC, and MC] showed a slowly rising trend with the growth of rice, and they were significantly higher than those of other treatments, but the EC of MI slowly decreased, and it was significantly lower than those of other treatments with NaCl. There was basically no difference in soil EC of RS, US, MS, RI, and UI treatments during rice growing. The EC of CK was the smallest and significantly smaller than those of the treatments with NaCl in the rice growth period.

### 3.2 Effects of different irrigation and N fertilization treatments on the Na^+^, K^+^, Ca^2+^, and Mg^2+^ contents of sea rice

It can be seen from Table 1 that different irrigation and N fertilization treatments have significant effects on Na^+^, K^+^, Ca^2+^, and Mg^2+^ contents of sea rice at tillering and booting stages. The Na^+^ content in plants of all treatments increased significantly with the growth of sea rice. The Na^+^ content of I (RI, UI, and MI) treatment was less than those of other treatments with NaCl, and that of S (RS, US, and MS) treatment was less than that of C treatment. The Na^+^ content of MI was significantly smaller than that of other treatments with NaCl, and that of CK was the smallest and significantly smaller than that of the treatments with NaCl.

**Table 1:** The Na^+^, K^+^, Ca^2+^, and Mg^2+^ contents of single plant under different irrigation and N fertilization treatments [mg/kg]

Treatments	Tillering stage				Booting stage			
	Na^+^	K^+^	Ca^2+^	Mg^2+^	Na^+^	K^+^	Ca^2+^	Mg^2+^
RS	3.58 ± 0.37	3.18 ± 0.38	1.41 ± 0.09	1.39 ± 0.06	7.08 ± 0.58	1.79 ± 0.08	0.67 ± 0.04	1.44 ± 0.12
US	4.40 ± 0.41	3.36 ± 0.52	1.47 ± 0.06	1.31 ± 0.12	6.61 ± 0.57	2.13 ± 0.15	0.60 ± 0.01	1.45 ± 0.19
MS	2.34 ± 0.33	4.14 ± 0.62	1.38 ± 0.11	1.31 ± 0.10	5.29 ± 0.42	4.05 ± 0.17	0.84 ± 0.07	1.38 ± 0.08
RI	3.52 ± 0.36	4.17 ± 0.29	1.57 ± 0.05	1.38 ± 0.11	5.41 ± 0.27	2.35 ± 0.26	0.67 ± 0.05	1.26 ± 0.13
UI	3.19 ± 0.22	3.46 ± 0.40	1.58 ± 0.11	1.24 ± 0.11	6.11 ± 0.31	2.83 ± 0.13	0.85 ± 0.02	1.54 ± 0.08
MI	1.59 ± 0.11	5.47 ± 0.18	1.61 ± 0.09	1.66 ± 0.15	4.90 ± 0.25	4.73 ± 0.29	1.00 ± 0.06	1.71 ± 0.15
RC	8.41 ± 0.64	2.28 ± 0.28	1.33 ± 0.07	1.26 ± 0.11	10.63 ± 0.84	1.56 ± 0.19	0.29 ± 0.03	1.22 ± 0.15
UC	5.52 ± 0.57	3.11 ± 0.16	1.00 ± 0.21	1.18 ± 0.16	9.38 ± 0.92	1.39 ± 0.09	0.38 ± 0.02	1.34 ± 0.12
MC	4.43 ± 0.14	2.95 ± 0.48	1.25 ± 0.05	1.25 ± 0.12	9.22 ± 0.40	1.38 ± 0.08	0.17 ± 0.02	1.22 ± 0.07
CK	0.82 ± 0.05	11.26 ± 1.66	1.46 ± 0.10	1.56 ± 0.13	2.89 ± 0.29	9.31 ± 0.06	0.95 ± 0.11	1.55 ± 0.15

Note: Numbers in the table are mean values ± standard deviation. The sea rice all died by salt stress at maturity stage, so the data at maturity stage is not showed.

The K^+^ and Ca^2+^ contents in plants of all treatments decreased significantly with the growth of sea rice. The K^+^ and Ca^2+^ contents of I were higher than those of S and C, and those of S were higher than those of C. The K^+^ content of MI was significantly higher than that of other treatments with NaCl, and the K^+^ content of CK was significantly higher than that of treatments with NaCl. The K^+^ content of MS was significantly higher than that of RS, US, RI, UI, and C, and the Ca^2+^ content of MI and CK was significantly higher than that of other treatments in rice booting stage.

The Mg^2+^ content of all treatments had no meaningful change during rice growth period. The Mg^2+^ content of MI and CK was significantly higher than that of other treatments in rice tillering stage. The Mg^2+^ content of MI was significantly higher than that of other treatments in rice booting stage.

### 3.3 Effects of different irrigation and N fertilization treatments on the CMP and MDA contents of sea rice

As shown in Table 2, the CMP and MDA contents of MI were significantly lower than those of other treatments with NaCl, and those of CK were significantly lower than those of the treatments with NaCl in the tillering stage of sea rice. In rice booting stage, the CMP of MI and UI was significantly less than that of RI and S, and that of RI and S was significantly lower than that of C, and that of CK was the smallest. The MDA content of I, S, and CK was significantly lower than that of C.

**Table 2:** The CMP and MDA contents of rice leaves under different irrigation and N fertilization treatments

Treatments	Tillering stage		Booting stage	
	CMP [%]	MDA [μmol/g FW]	CMP [%]	MDA [μmol/g FW]
RS	31.90 ± 0.16	1.09 ± 0.15	40.12 ± 0.82	1.31 ± 0.12
US	31.53 ± 0.35	1.04 ± 0.12	38.64 ± 0.51	1.11 ± 0.12
MS	31.67 ± 0.58	1.00 ± 0.09	39.08 ± 0.99	1.00 ± 0.10
RI	30.24 ± 2.09	1.17 ± 0.03	40.83 ± 0.89	1.23 ± 0.21
UI	30.29 ± 0.41	0.99 ± 0.06	34.17 ± 0.95	1.00 ± 0.12
MI	26.14 ± 1.77	0.73 ± 0.08	32.82 ± 0.03	1.05 ± 0.11
RC	31.33 ± 0.11	1.32 ± 0.04	72.28 ± 2.17	1.43 ± 0.21
UC	31.52 ± 1.08	1.18 ± 0.13	77.10 ± 0.17	1.66 ± 0.12
MC	31.56 ± 1.34	1.14 ± 0.12	79.71 ± 0.94	1.62 ± 0.20
CK	12.66 ± 3.49	0.88 ± 0.22	13.70 ± 4.13	0.99 ± 0.18

Note: Numbers in the table are mean values ± standard deviation. The sea rice all died by salt stress at maturity stage, so the data at maturity stage is not showed.

### 3.4 Effects of different irrigation and N fertilization treatments on the plant height of sea rice

**Figure 2:**
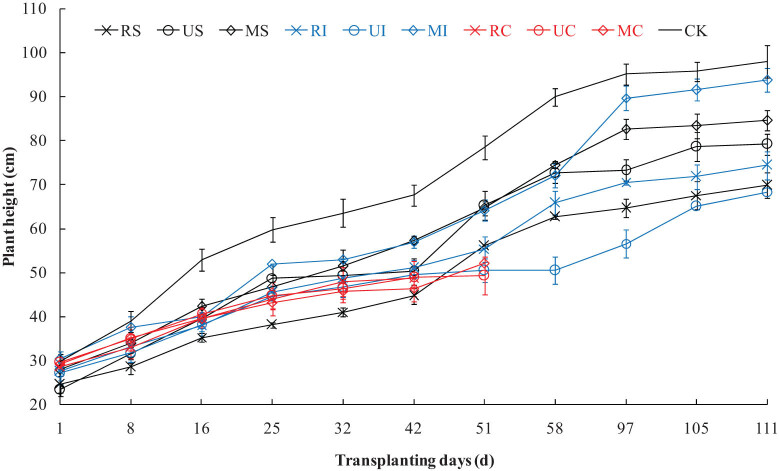
The plant height of sea rice under different irrigation and N fertilization treatments.

As shown in Figure 2, the rice height of each treatment gradually increased with the growth of sea rice; however, the sea rice of C grew slowly after 32 days, and the sea rice of RC died at 51 days, and that of UC and MC also died at 58 days. The plant height of CK was significantly higher than that of other treatments during the whole growth period of rice. After 58 days, the plant height of MI was significantly higher than that of other treatments with NaCl, and that of MS was significantly higher than that of US, RS, RI, and UI, and that of US was significantly higher than that of RS, RI, and UI, and that of RI was significantly higher than that of RS and UI, and that of RS was significantly higher than that of UI.

### 3.5 Effects of different irrigation and N fertilization on the nitrogen uptake of single plant

As shown in Table 3, the nitrogen uptake of rice treated with S, I, and CK increased significantly with the growth of rice, and the nitrogen uptake of rice of C was smaller than that of other treatments, and all rice of C died without nitrogen accumulation at maturity stage. The nitrogen uptake of MI and CK was significantly higher than that of other treatments at tillering stage. The order of rice nitrogen accumulation of treatments was CK, MI > US, MS > RI, UI > RS, RC > UC, MC at booting stage. The nitrogen uptake of CK was significantly greater than that of treatments with NaCl, and that of MI was significantly greater than that of other treatments with NaCl at maturity stage, 19% more than UI, 36% more than MS, 56% more than US, 75% more than RI, and 86% more than RS, respectively.

**Table 3:** The nitrogen uptake of single plant under different irrigation and N fertilization treatments

Treatments	Tillering stage	Booting stage	Mature stage
RS	5.02 ± 0.85	28.79 ± 2.17	335.89 ± 20.70
US	16.26 ± 1.20	95.02 ± 6.19	400.17 ± 19.78
MS	10.06 ± 0.83	90.64 ± 2.55	458.86 ± 11.25
RI	7.50 ± 0.69	63.36 ± 2.32	356.20 ± 26.57
UI	8.18 ± 1.56	78.51 ± 8.88	524.68 ± 17.58
MI	21.45 ± 2.44	114.49 ± 15.88	623.45 ± 14.37
RC	9.07 ± 0.36	32.94 ± 3.28	/
UC	11.39 ± 1.05	21.68 ± 2.31	/
MC	11.87 ± 1.44	18.41 ± 3.39	/
CK	21.28 ± 1.31	122.85 ± 15.06	731.20 ± 41.51

Note: Numbers in the table are mean values ± standard deviation. “/” means that the sea rice all died by salt stress at maturity stage.

### 3.6 Effects of different irrigation and N fertilization treatments on the dry weight of sea rice

**Figure 3:**
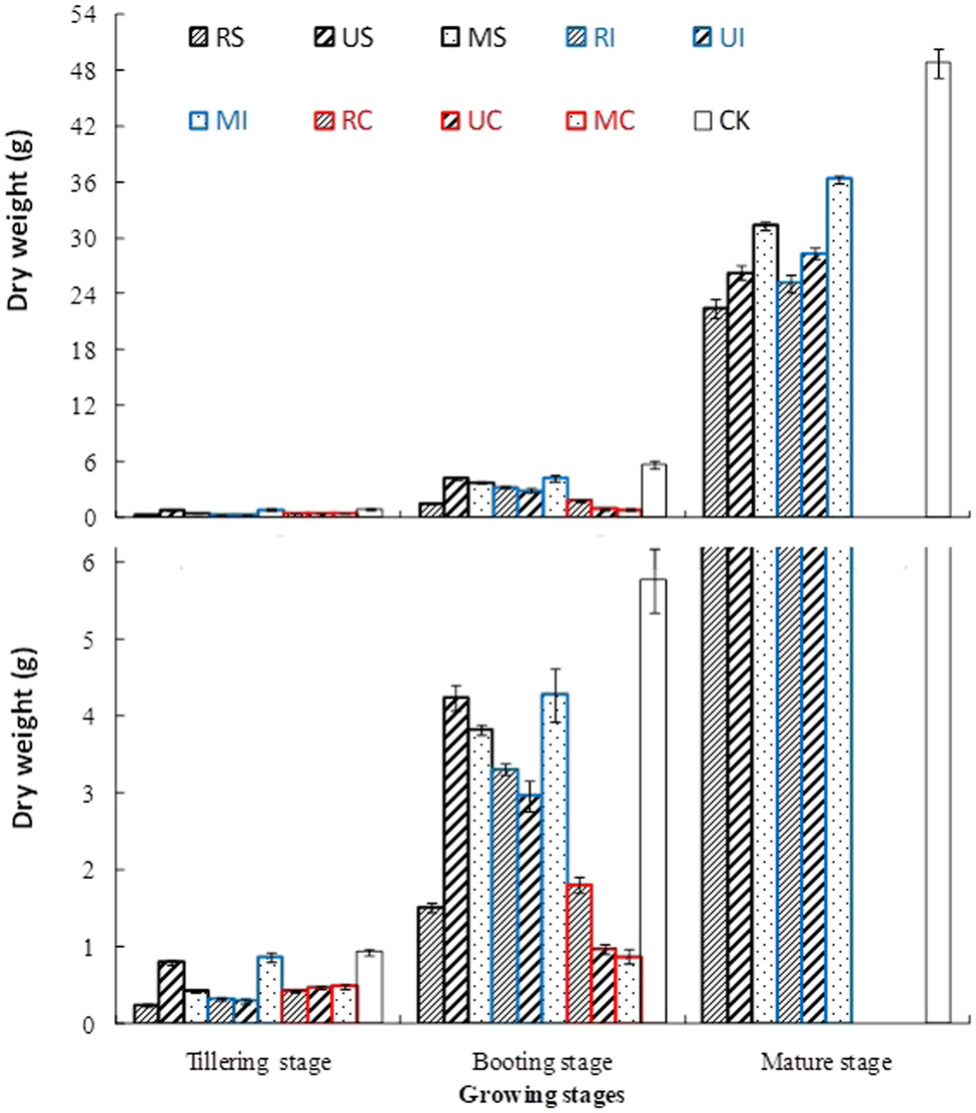
Effects of different irrigation and N fertilization treatments on the dry weight of sea rice.

As shown in Figure 3, the dry weight of rice of S, I, and CK increased significantly with rice growing, while the rice of C grew slower than that of other treatments, and all of them died at maturity stage. The rice dry weight of US, MI, and CK was significantly higher than that of other treatments at tillering stage. The order of rice dry weight of treatments was CK > US, MI > MS > RI, UI > RS, RC > UC, MC at booting stage. In maturity stage, the rice dry weight of CK was significantly higher than that of treatments with NaCl, that of MI was significantly higher than that of other treatments with NaCl, 16% higher than MS, 28% higher than UI, 38% higher than US, 44% higher than RI, and 61% higher than RS, respectively.

### 3.7 Effects of different irrigation and N fertilization treatments on the yield of sea rice

**Figure 4:**
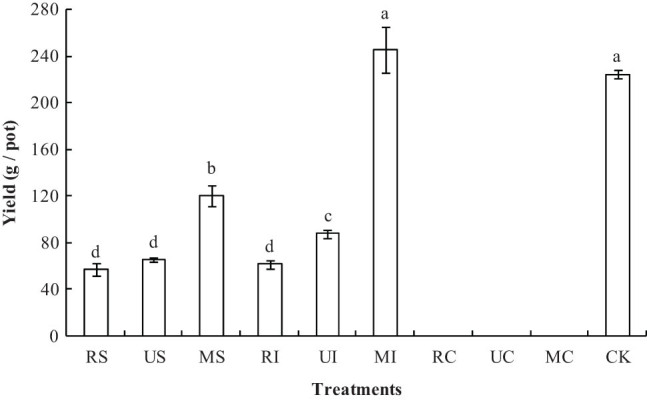
Effects of different irrigation and N fertilization treatments on the yield of sea rice. Note: Different lower-case letters in a column indicate significant difference among treatments at the 5% level.

It can be seen from Figure 4 that the effects of different irrigation and N fertilization treatments on the yield of sea rice are remarkable. The rice yield of MS was significantly higher than that of RS and US, and the rice yield of MI was also significantly higher than that of RI and UI. The sea rice treated with C had all died at maturity, so they had almost no yield. There was no difference in yield between MI and CK, and the yield of MI and CK were significantly higher than those of other treatments. The yield of MI was 104% higher than that of MS, 108% higher than that of UI, 277% higher than that of US, 300% higher than that of RI, and 334% higher than that of RS, respectively.

## 4 Discussion

Soil EC is closely related to soil salinity, and there is a linear relationship between them [17], so the EC can better reflect soil salinity. Because the irrigation amount of S and I was more than that of C, the salt in upper layer of soil was diluted, and the salt ions (Na^+^, Cl^−^, etc.) were leached below root layer soil [18]. The irrigation amount of C was less, which was 80–100% of the soil field capacity. The salt ions could not be fully leached to the lower soil, and they converged to the upper soil with water evaporating rapidly, resulting in the increase in EC and Na^+^ content in root layer soil [19]. Therefore, the EC in root layer soil of S and I was significantly lower than that of C. The root layer soil EC of MI was significantly lower than that of other treatments with NaCl, which was because the soluble salt was leached to the lower layer soil by the intermittent irrigation method (I) [18], and the mixed fertilization of controlled release urea and urea (M) was beneficial to the nitrogen absorption and growth of sea rice in the whole rice growth period, so the biomass of sea rice treated with MI was larger, and the nutrients of rice such as NH_4_^+^, PO_4_^3−^, K^+^, Ca^2+^, and Mg^2+^ were absorbed more from the soil [20]. At the same time, the EC of root layer soil was low by controlling nitrogen release.

Soil salinization is a major environmental salt stress, which increases the ionic toxicity and osmotic stress of plants, resulting in the decline of plant growth and function. The adverse effects of salinity are usually related to the imbalance of plant nutrition caused by excessive uptake of Na^+^ or Cl^−^ [21]. This study showed that the EC (mainly NaCl) in root layer soil of C, S, and I treatments was significantly reduced in order, and that of MI was the smallest. Moreover, the Na^+^ content of the plant treated with C, S, and I also decreased significantly in order, and that of MI was the lowest. So the Na^+^ content in root layer soil was positively correlated with the Na^+^ content of rice, because the high content of Na^+^ in the rhizosphere soil led to the absorption of Na^+^ by rice root cells through K^+^ transporters, while the absorption of K^+^ decreased [21]. The results of this study also verified that the K^+^ and Ca^2+^ contents in sea rice of I were higher than those of S and C, and the K^+^, Ca^2+^, and Mg^2+^ contents in rice of MI were significantly higher than those of other treatments with salt stress. Therefore, Na^+^ stress has a negative impact on plant nutrient uptake, resulting in plant exposure to osmotic stress and affecting the content of other specific nutrients in plant. Due to nutrient imbalance, crop yield and quality under salinity stress will decline [22]. This study showed that the sea rice treated with C had the highest Na^+^ content and damaged most seriously by Na^+^ toxicity, resulting in slow growth and death of the rice. The plant height and dry weight of MI were significantly higher than other treatments with NaCl, this is because the content of K^+^, Ca^2+^, and Mg^2+^ in the sea rice treated with MI are higher, and higher K^+^/Na^+^, Ca^2+^/Na^+^, and Mg^2+^/Na^+^ in rice play an important role in mitigating salt-induced Na^+^ toxicity [23]. Plants can avoid the decrease in K^+^/Na^+^ and Ca^2+^/Na^+^ by reducing Na^+^ into cells, removing Na^+^ from cells and separating Na^+^ into vacuoles, so as to maintain ionic stability and avoid damaging any cell function [24].

CMP can reflect the integrity and damage degree of cell membrane in rice leaves [25]. MDA is the final decomposition product of cell membrane lipid peroxidation, and its content can indirectly reflect the damage degree of cell membrane [26]. Generally, the greater the damage for rice under salt stress, the higher the CMP and MDA contents in rice leaves [27]. This study showed that the MDA content of I and S were significantly lower than that of C, and the CMP and MDA contents of MI and CK were significantly lower than those of other treatments. This is closely related to the Na^+^ content of sea rice. The Na^+^ content in rice of MI and CK is lower, the intracellular ions are more balanced, and the Na^+^ toxicity for rice is lighter, so, CMP and MDA contents in rice leaves are smaller. Similarly, the Na^+^ content of sea rice treated with I and S was lower than that of C, while the Ca^2+^ content was higher, and the Na^+^ toxicity for the sea rice of I and S is lighter, so the MDA content of I and S was lower than that of C. According to the growth status and nutrients content change in sea rice treated with I, S, and C, the Na^+^ content in plants increased significantly, while the K^+^ and Ca^2+^ contents decreased significantly with sea rice growing. Therefore, the sea rice was gradually damaged by Na^+^ toxicity, and the Na^+^ content of sea rice treated with C was the highest, finally they all died at maturity.

The stunted growth and biomass decrease in plant is one of the main consequences of salt stress, which usually reduces the yield of most plant [6]. In this study, the sea rice yield of I and S was significantly higher than that of C, and that of M was significantly higher than that of R and U. This is because different irrigation methods may significantly impact rice photosynthesis and respiration by altering the movement of water, ions (Na^+^, K^+^, Ca^2+^, and Mg^2+^) and organic solutes across the cell [28], and the Na^+^ toxicity to sea rice of I and S is lighter than that of C. Moreover, the nitrogen supply law of mixed fertilization treatment (M) was consistent with the nitrogen demand law of sea rice, and the nitrogen for rice could be supplied by urea on the early rice growth stage, and the nitrogen could be supplied by controlled-release urea on the middle and late rice growth stage [29]. Nitrogen is the most yield-restraining nutrient in crop production globally. Efficient nitrogen fertilization is one of the most important factors for improving nitrogen use efficiency, field crops productivity, and profitability [30]. Good nitrogen nutrition played an important role in promoting the growth and yield of rice [31], so the yield of rice treated with M was significantly higher than that treated with C and U. Because the treatment with MI was beneficial to the nitrogen uptake of sea rice, mitigate salt-induced Na^+^ toxicity, and sustain sea rice growth, the yield of sea rice of MI was the largest. we suggested that the treatment of MI should be adopted for planting sea rice on the local study areas, which is beneficial to mitigate Na^+^ toxicity and increase yield of sea rice. This study is based on the greenhouse simulation experiment, which has the experimental condition like local field production [salinity of saline-sodic soil, local sea rice, fertilization, irrigation, climate, etc.], so this experiment has important reference for local sea rice planting. However, actual rice field production conditions are often not fully controlled, it is necessary to study the effects of irrigation and fertilization on mitigating Na^+^ toxicity and maintaining sea rice growth under salinity stress by field experiments in further research.

## 5 Conclusion

The irrigation and N fertilization have a significant impact on the root layer soil EC and Na^+^, K^+^, Ca^2+^, and Mg^2+^ contents of sea rice. The EC of MI was significantly smaller than that of other treatments with NaCl, and the Na^+^ content of MI was the lowest, while the K^+^, Ca^2+^, and Mg^2+^ contents of MI were the highest in salt treatments. The MDA and CMP of MI were significantly smaller than those of other treatments with NaCl. On the maturity stage of rice, the nitrogen uptake and dry weight of MI were the highest in treatments with NaCl, and the rice yield of MI was significantly larger than that of other treatments with NaCl, and was increased by 104, 108, 277, 300, and 334% compared with MS, UI, US, RI, and RS, respectively. Therefore, we believe that the treatment of mixed fertilization integrating intermittent irrigation (MI) can be used to plant sea rice on local saline-sodic soil. The MI treatment can effectively reduce Na^+^ content, while increase K^+^/Na^+^ and Ca^2+^/Na^+^ of rice, and mitigate salt-induced Na^+^ toxicity to sea rice, promote the N absorption and biomass accumulation of rice, and raise the yield of sea rice.

## References

[1] Liang W, Ma X, Wan P, Liu L. Plant salt-tolerance mechanism: a review. Biochem. Biophys Res Commun. 2018;495:286–91. 10.1016/j.bbrc.2017.11.043 29128358

[2] Vinocur B, Altman A. Recent advances in engineering plant tolerance to abiotic stress: achievements and limitations. Curr Op Biotechnol. 2005 Apr 1;16(2):123–32. 10.1016/j.copbio.2005.02.001 15831376

[3] Chen YS, Wang P, Wang KX, Chen N, Zhao L. The strategic choice of sea rice industry development in China. J Ocean U China. 2018;1:50–4.

[4] Hong LZ, Liu XH, Wang MW. The saline soil agricultural technology in coastal area of Jiangsu. Nanjing: Nanjing University Press; 2015. p. 1–17 (in Chinese).

[5] Zhu JK. Plant salt tolerance. Trends Plant Sci. 2001;6:66–71. 10.1016/s1360-1385(00)01838-0 11173290

[6] Khalifa GS, Abdelrassoul M, Hegazi AM, Elsherif MH. Attenuation of negative effects of saline stress in two lettuce cultivars by salicylic acid and glycine betaine. Gesunde Pflanz. 2016 Dec;68(4):177–89.

[7] Parida AK, Das AB. Salt tolerance and salinity effects on plants: a review. Ecotoxicol Environ Saf. 2005 Mar 1;60(3):324–49. 10.1016/j.ecoenv.2004.06.010 15590011

[8] Lima GS, Pinheiro FW, Gheyi HR, Soares LA, Silva SS. Growth and post-harvest fruit quality of West Indian cherry under saline water irrigation and potassium fertilization. Rev Caatinga. 2020;33:775–84.

[9] Kanawapee N, Sanitchon J, Srihaban P, Theerakulpisut P. Physiological changes during development of rice [Oryza sativa L.] varieties differing in salt tolerance under saline field condition. Plant Soil. 2013 Sep;370(1):89–101.

[10] Zhu CL, Zhang ZY. Effects of irrigation mode on nitrogen and phosphorus loss and environment in paddy field. Water Resources Protection. 2013;19(6):56–8.

[11] Qi H, Ma R, Shi C, Huang Z, Liu S, Sun L, et al. Novel low-cost carboxymethyl cellulose microspheres with excellent fertilizer absorbency and release behavior for saline-alkali soil. Int J Biol Macromol. 2019;131:412–9. 10.1016/j.ijbiomac.2019.03.047 30853583

[12] Zhang Y, Fang J, Wu X, Dong L. Na+/K+ balance and transport regulatory mechanisms in weedy and cultivated rice [Oryza sativa L.] under salt stress. BMC Plant Biol. 2018;18(1):375. 10.1186/s12870-018-1586-9 PMC6311050 30594151

[13] Huang LH, Liang ZW, Suarez DL, Wang ZC, Wang MM, Yang HY, et al. Impact of cultivation year, nitrogen fertilization rate and irrigation water quality on soil salinity and soil nitrogen in saline-sodic paddy fields in Northeast China. J Agric Sci. 2016 May;154(4):632–46.

[14] Lu RL. Soil agricultural chemical analysis method. Beijing: China Agricultural Science and Technology Press; 2000. p. 1–315.

[15] Xiang S, Ma X, Shi H, Ma T, Tian C, Chen Y, et al. Green synthesis of an alginate-coated silver nanoparticle shows high antifungal activity by enhancing its cell membrane penetrating ability. ACS Appl Bio Mater. 2019;2(9):4087–96. 10.1021/acsabm.9b00590 35021342

[16] Qu KC, Wang ZY, Tang KK, Zhu YS, Fan RF. Trehalose suppresses cadmium-activated Nrf2 signaling pathway to protect against spleen injury. Ecotoxicol Environ Saf. 2019;181:224–30. 10.1016/j.ecoenv.2019.06.007 31195231

[17] Lv GJ, Yuan QL, Liu XG. Research on the influencing factors of soil electrical conductivity in saline soil. China Rural Water Hydropower. 2020;457:124–6 (in Chinese).

[18] Hu YT, Gao J. The dynamic changes of soil salinity in different growth periods of rice growing along the Yellow river in Shaanxi province under well water irrigation. LDev Eng Res. 2018;3:61–6 (in Chinese).

[19] Sarwar G, Schmeisky H, Hussain N, Malik MA, Manzoor MZ, Zafar A, et al. Impact of compost to produce rice-wheat crops from saline sodic soil. J Pure Appl Agriculture. 2020;5(1):11–9.

[20] Manohara KK, Morajkar S, Shanbagh Y. Genetic analysis of grain yield and its associated traits in diverse salt-tolerant rice genotypes under coastal salinity condition. J Cereal Res. 2020;12:290–6.

[21] Hamani AK, Chen J, Soothar MK, Wang G, Shen X, Gao Y, et al. Application of exogenous protectants mitigates salt-induced Na+ toxicity and sustains cotton [Gossypium hirsutum L.] seedling growth: Comparison of glycine betaine and salicylic acid. Plants. 2021;10:380. 10.3390/plants10020380 PMC7923183 33671193

[22] Awal MA, Ikeda T. Recovery strategy following the imposition of episodic soil moisture deficit in stands of peanut [Arachis hypogaea L.]. J Agron Crop Sci. 2002;188(3):185–92.

[23] Jin J, Cui H, Lv X, Yang Y, Wang Y, Lu X. Exogenous CaCl2 reduces salt stress in sour jujube by reducing Na+ and increasing K+, Ca2+, and Mg2+ in different plant organs. J Horticultural Sci Biotechnol. 2017;92:98–106.

[24] Yildirim E, Ekinci M, Turan M, Dursun A, Kul R, Parlakova F. Roles of glycine betaine in mitigating deleterious effect of salt stress on lettuce [Lactuca sativa L.]. Arch Agron Soil Sci. 2015;61:1673–89.

[25] Ran C, Gulaqa A, Zhu J, Wang X, Zhang S, Geng Y, et al. Benefits of biochar for improving ion contents, cell membrane permeability, leaf water status and yield of rice under saline–sodic paddy field condition. J Plant Growth Regul. 2020 Mar;39(1):370–7.

[26] Fu X, Ma L, Gui R, Ashraf U, Li Y, Yang X, et al. Differential response of fragrant rice cultivars to salinity and hydrogen rich water in relation to growth and antioxidative defense mechanisms. Int J Phytoremediat. 2021;23:1203–11. 10.1080/15226514.2021.1889963 33617358

[27] Al-Zahrani HS, Alharby HF, Fahad S. Antioxidative defense system, hormones, and metabolite accumulation in different plant parts of two contrasting rice cultivars as influenced by plant growth regulators under heat stress. Front Plant Sci. 2022;13:911846. 10.3389/fpls.2022.911846 PMC9196032 35712584

[28] Fahad S, Adnan M, Hassan S, Saud S, Hussain S, Wu C, et al. Rice responses and tolerance to high temperature. Advances in rice research for abiotic stress tolerance. UK: Woodhead Publishing; 2019. p. 201–24.

[29] Ye Y, Liang X, Chen Y, Liu J, Gu J, Guo R, et al. Alternate wetting and drying irrigation and controlled-release nitrogen fertilizer in late-season rice. Effects on dry matter accumulation, yield, water and nitrogen use. Field Crop Res. 2013;144:212–24.

[30] Amanullah K, Fahad S, editors. Nitrogen in agriculture: Updates. BoD–Books on Demand. Hamburg; 2018.

[31] Liu Y, Wang H, Jiang Z, Wang W, Xu R, Wang Q, et al. Genomic basis of geographical adaptation to soil nitrogen in rice. Nature. 2021;590:600–5. 10.1038/s41586-020-03091-w 33408412

